# Epstein-Barr virus: the mastermind of immune chaos

**DOI:** 10.3389/fimmu.2024.1297994

**Published:** 2024-02-07

**Authors:** Jean de Melo Silva, Carlos Eduardo de Castro Alves, Gemilson Soares Pontes

**Affiliations:** ^1^ Laboratory of Virology and Immunology, National Institute of Amazonian Research (INPA), Manaus, AM, Brazil; ^2^ Post-Graduate Program in Basic and Applied Immunology, Institute of Biological Science, Federal University of Amazonas, Manaus, AM, Brazil

**Keywords:** EBV, herpesvirus, evasion, innate immunity, acquired immunity

## Abstract

The Epstein-Barr virus (EBV) is a ubiquitous human pathogen linked to various diseases, including infectious mononucleosis and multiple types of cancer. To control and eliminate EBV, the host’s immune system deploys its most potent defenses, including pattern recognition receptors, Natural Killer cells, CD8^+^ and CD4^+^ T cells, among others. The interaction between EBV and the human immune system is complex and multifaceted. EBV employs a variety of strategies to evade detection and elimination by both the innate and adaptive immune systems. This demonstrates EBV’s mastery of navigating the complexities of the immunological landscape. Further investigation into these complex mechanisms is imperative to advance the development of enhanced therapeutic approaches with heightened efficacy. This review provides a comprehensive overview of various mechanisms known to date, employed by the EBV to elude the immune response, while establishing enduring latent infections or instigate its lytic replication.

## 1 Introduction

Epstein-Barr virus (EBV) is a highly prevalent human herpesvirus worldwide, disseminated in 90-95% of the adult population (1–3). This ubiquitous virus was first identified in 1964 by Anthony Epstein and his team. Their discovery arose from observing herpesvirus-like particles in cultured tumor cells from African Burkitt lymphoma, a childhood cancer prevalent in Africa (4).

EBV belongs to the *Herpesviridae* family, *Gammaherpesvirinae* subfamily and is classified as human herpesvirus 4 (HHV-4) (5). It is an enveloped icosahedral virus, with an approximate size of 150nm in diameter and has a capsid formed by 162 capsomers (6). Its viral genome consists of a linear double-stranded DNA molecule of 172 kbp, which encodes approximately 100 viral proteins (6). EBV is mainly transmitted through direct contact with the saliva of infected individuals, such as kissing, sharing eating or drinking utensils. It can also be transmitted through blood transfusions and organ transplants (7).

EBV has tropism for epithelial cells of the nasopharynx and oropharynx, as well as for B lymphocytes (8). Epithelial cells are infected when viral glycoproteins gHgL and gB interact with certain integrins (αvβ5, αvβ6, or αvβ8). B lymphocytes are infected when the viral protein gp350/220 binds to the cellular protein CD21. This is followed by the binding of three other viral proteins (gp85, gp25, and gp42) to the class II human leukocyte antigen (HLA II) (9, 10). In both cases, EBV can enter the host cell by endocytosis or fusion with the plasma membrane (8).

After insertion of the EBV genome into the cell nucleus, the virus can start its lytic cycle or enter a latency state, each with expression of different genes (11). The lytic phase begins with the expression of the *BZLF-1* and *BRLF-1* genes, which encode the transactivating proteins Zta and Rta, respectively, responsible for the course of the lytic phase of EBV (12). This cycle has three phases (early-immediate, early, and late) and generally occurs in epithelial cells, where virions are produced and the host cell is lysed (13, 14). In individuals with latent EBV infection, the lytic cycle can occur in tonsil plasma cells. These cells are mature B lymphocytes specialized in producing antibodies, also known as immunoglobulins (15).

During the latent phase, EBV exists as an episome in the nucleus of host cells, especially B cells. This phase is divided into three latency forms (I, II, and III) and involves the expression of six nuclear antigens (EBNAs), two latent membrane proteins (LMPs), and non-coding RNAs (EBERs) (14). In the latency phase, EBV persists in the body using complex mechanisms to evade detection and elimination by the immune system, without causing symptoms (11). However, EBV can be reactivated in situations such as immunosuppression or stress (16).

EBV infection is associated with a variety of diseases, including infectious mononucleosis (IM), lymphomas (such as Burkitt lymphoma, Hodgkin lymphoma, and B-cell lymphomas in immunocompromised patients), some types of epithelial cell cancers (such as nasopharyngeal carcinoma), and certain types of gastric cancer (17–22). Evidence also suggests a link between EBV infection and certain autoimmune diseases, such as Sjögren syndrome and systemic lupus erythematosus, but the exact mechanisms of this relationship are still unknown (23).

EBV has perplexed scientists for decades. Its masterful ability to evade immune surveillance and establish persistent latent infections, while simultaneously contributing to a diverse range of diseases, remains a source of awe and intrigue. Despite relentless scrutiny, many aspects of EBV’s dynamic interplay with the host’s immune system remain shrouded in mystery. This comprehensive review aims to shed light on the intricate mechanisms through which EBV orchestrates its immune evasion strategies, highlighting recent advances in this burgeoning field. Additionally, it will identify critical lacunae within the current body of knowledge compelling us to embark on investigative journeys to unravel the enigmas that persist.

## 2 The elusive escape artist: unraveling EBV’s art of immune evasion

EBV stands as a maestro of immune subversion, orchestrating a complex symphony of multifaceted mechanisms to compose its enduring sojourn within the host’s domain— a mesmerizing ballet of evasion akin to an eternal pas de deux. A prevailing hypothesis posits that a substantial proportion, exceeding 50%, of the proteins encoded by EBV’s genomic constituents are intricately involved in effectuating evasion from host immune responses via a nuanced modulation of the immune machinery (24, 25). Below, a panoramic overview reveals the captivating dance of escape, a balletic tapestry woven by EBV’s deft artistry.

### 2.1 EBV’s masterful manipulation of cell death

Apoptosis, or programmed cell death, is a cellular defense mechanism that can protect the body from infection. However, some viruses such as EBV have evolved mechanisms to inhibit apoptosis, which allows them to replicate and spread to new cells (26). EBV latent membrane proteins 1 and 2 (LMP1 and LMP2) are expressed in several types of cancer and interfere in several cell characteristics, such as cell signaling, differentiation, migration and growth, being therefore involved in tumorigenicity (27).

A recent study revealed the significant impact of EBV on B cell differentiation in the context of diffuse large B cell lymphoma (DLBCL) (28). EBV activates the gene *HLX* through the molecular conduits LMP1 and LMP2A, using the signal transducer and activator of transcription 3 (STAT3) pathway. The heightened activity of the *HLX* gene has a dual impact on key cellular processes. Firstly, it represses the expression of the pro-apoptotic gene *BCL2L11*, responsible for encoding the Bim protein—a crucial member of the Bcl-2 family. Bim protein plays a vital role in the cell death process. Additionally, the overexpression of *HLX* leads to the suppression of the *IL4R*, *NKX6-3*, and *SPIB* genes, all of which contribute to plasma cell differentiation. In the context of DLBCL, the inhibition of the *BCL2L11* gene facilitates the survival of infected B cells. This survival mechanism is pivotal in the progression of DLBCL. Simultaneously, the inhibition of *IL4R*, *NKX6-3*, and *SPIB* genes impedes B cell differentiation (28). These molecular events collectively highlight potential therapeutic targets for intervention in DLBCL. Understanding and manipulating these specific genetic pathways could offer novel approaches to combat this disease.

Similarly, the EBV-encoded *BHRF1* gene, expressed during latency, exhibits anti-apoptotic activity and contributes to chemoresistance. Its functional similarity to the cellular Bcl-2 protein allows BHRF1 to directly suppress apoptosis induced by anticancer drugs (29). Furthermore, BHRF1 sequesters pro-apoptotic proteins like Bim, Bid, Puma, and Bak, further inhibiting DNA damage-induced cell death (30, 31). These findings position *BHRF1* as a potential therapeutic target for EBV-associated malignancies.

**Figure 1:**
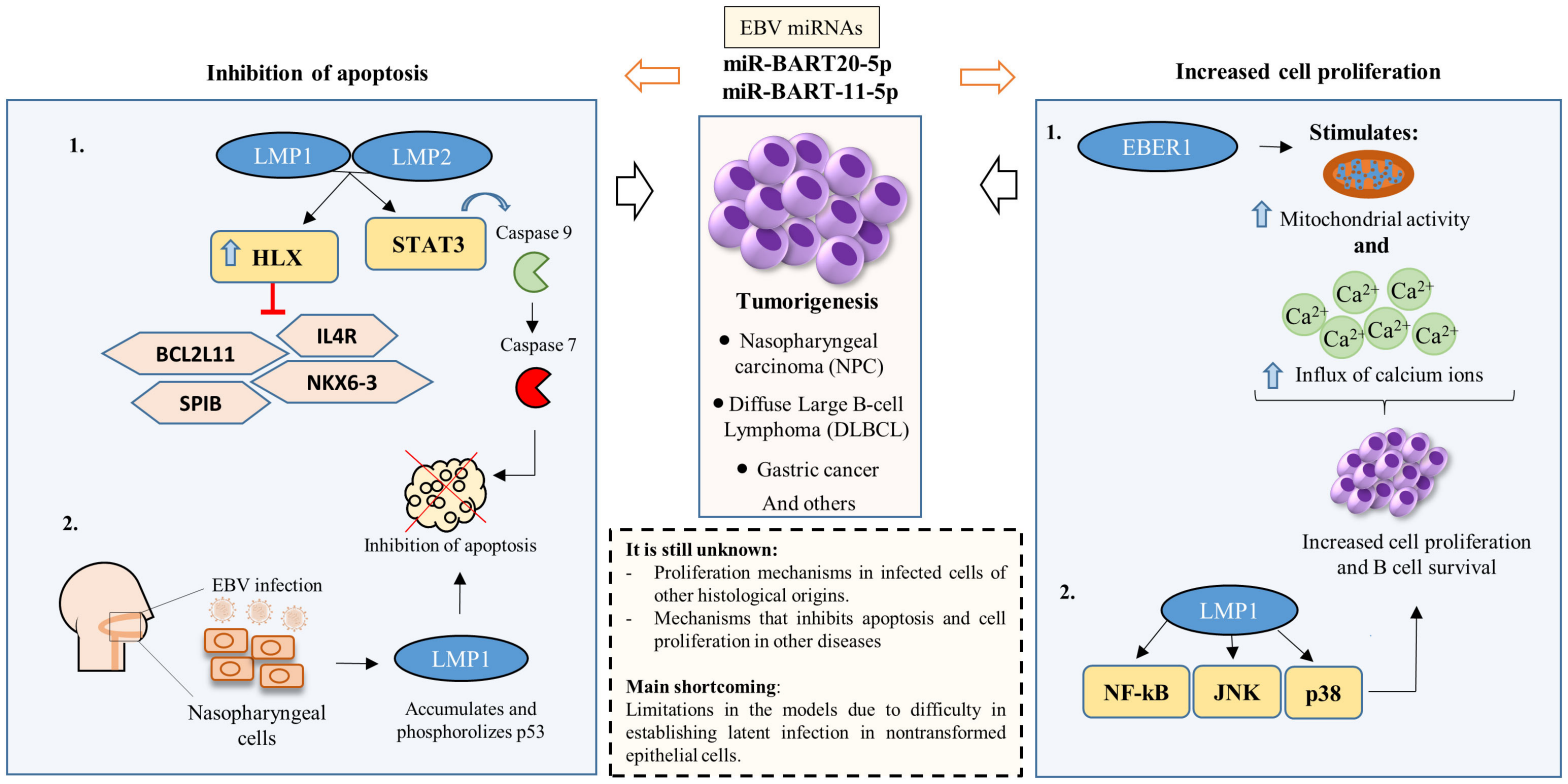
EBV’s impact on apoptosis inhibition and the stimulation of infected cell proliferation. Apoptosis inhibition primarily occurs via two mechanisms (1): Latency proteins LMP1 and LMP2 modulate various molecules and transcriptional pathways, suppressing host cell apoptosis to facilitate EBV latency persistence; and (2) Infection in nasopharyngeal epithelial cells prompts LMP1 accumulation and p53 phosphorylation, inhibiting apoptosis. Additionally, cell proliferation and B cell survival are predominantly induced by (1): EBER1 enhances mitochondrial activity and calcium influx; and (2) LMP1 induces different transcription factors associated with promoting cell proliferation and B cell survival. EBV miRNAs, particularly miR-BART20-5p and miR-BART11-5p, play a pivotal role in both inhibiting apoptosis and promoting cell proliferation.

Within the complex scenario of cellular dynamics, a latent cytoplasmic transcription factor, STAT3, assumes a versatile role. This enigmatic factor coordinates a symphony of vital cellular functions, encompassing respiration, regenerative processes, survival mechanisms, and the delicate balance of cell growth (32). It is this elaborate arrangement of activities that renders the activation of STAT3 a critical determinant in the genesis and progression of numerous cancers (33). The interplay is fascinating: EBV deftly invokes the activation of caspase 9 through STAT3, defying cellular fate by proficiently tipping the scales of transcript equilibrium, thus engineering the activation of caspase 7 and briskly evading apoptosis. Therefore, the role of STAT3 extends to triggering the emergence of the anti-apoptotic variant of caspase 9 within EBV-infected B lymphocytes (**Figure 1**) (34).

Despite the well-defined role of LMP1 in cell death regulation, the specific involvement of EBV microRNAs in this process remains largely enigmatic. This gap in knowledge necessitates a deeper exploration of these microRNAs, particularly their established anti-apoptotic properties and their potential contributions to the multifaceted immune evasion strategies employed by the virus. Therefore, the ensuing segment of this section aims to delineate the distinct mechanisms through which EBV microRNAs modulate cell death pathways and facilitate immune escape, offering insights into their potential impact on EBV-associated pathogenesis.

For about 20 years, non-coding RNAs have been investigated in EBV infection. It is now known that EBERs, for example, prevent EBV-infected cells from undergoing apoptosis induced by interferon alpha (35). The noncoding RNAs include the EBERs Epstein-Barr virus-encoded RNAs (EBERs), BamHI-A rightward transcripts (BARTs), EBV Small nucleolar RNAs (snoRNAs) and microRNAs (miRNAs) (36). EBV miRNAs are considered the first miRNAs identified in viruses (37). They specifically target both viral and cellular mRNAs, playing biological roles closely linked to the EBV replicative cycle (38). Studies that investigated the EBV non-coding RNAs identified two EBERs (EBER1 and EBER2) and more than 30 miRNAs (36, 39). Other more recent review studies identified that EBV encodes about 44-48 mature miRNAs that are involved in immune escape, cell proliferation and apoptosis, for example (40, 41).

In nasopharyngeal carcinoma (NPC), the expression of BART miRNAs has a wide range of consequences. These include suppressing cell apoptosis, promoting tumor metastasis, maintaining viral latency, facilitating cellular expansion and proliferation, and evading the immune system (40). Also, in NPC, p53 is accumulated and phosphorylated by EBV oncoprotein LMP1. Thus, LMP1 rescue tumor cell apoptosis and cell cycle (**Figure 1**) (42).

The EBV microRNA miR-BART20-5p is strongly associated with the invasive stage of nasal NK/T-cell lymphoma (NNL). It inhibits translation of the transcription factor T-bet, which in turn suppresses the tumor suppressor protein (43). T-bet plays an important role in both acquired and innate immunity by regulating the expression of cytokines, chemokines, cytokine receptors, and adhesion molecules, which influence the differentiation and development of immune cells, such as T and NK (44, 45). The p53 protein protects genomic integrity by preventing the growth and division of cells with damaged DNA (46). By suppressing p53, miR-BART20-5p contributes to the development of NNL.

Two EBV miRNAs, miR-BART20-5p and miR-BART11-5p, play important roles in the development of EBV-associated gastric carcinoma. miR-BART20-5p reduces apoptosis (programmed cell death) and increases cell growth, while miR-BART11-5p inhibits apoptosis and promotes proliferation and migration of gastric cancer cells (47). Both of these miRNAs contribute to tumorigenesis by altering the regulation of key cellular processes.

A study found that miR-BART15, which is secreted in exosomes from infected B cells, can inhibit the NLRP3 inflammasome in noninfected cells (48). This is an important strategy for EBV to evade the immune system, as the NLRP3 inflammasome plays a critical role in antiviral responses. Suppression of the inflammasome can lead to an immunosuppressive state, which allows EBV to persist and replicate in the host (49). Additional EBV-encoded miRNAs contribute to immune escape through diverse mechanisms. For instance, miR-BART4-3p exerts a multifaceted influence on gastric carcinoma cells by regulating proliferation, apoptosis, and migration (50). Similarly, miR-BART4-5p promotes immune evasion by downregulating proapoptotic proteins, thereby reducing apoptosis in gastric cancer cells (51).

One of the key mechanisms that EBV uses to evade the immune response is to prevent programmed cell death. While many studies have investigated the role of EBV proteins in this process, there is less research on the role of EBV microRNAs. This is a significant gap in knowledge, as the functions of most EBV microRNAs are still unknown. Therefore, more research is needed to elucidate the role of EBV microRNAs in immune evasion. This knowledge could be used to develop new therapeutic strategies for EBV-associated diseases.

### 2.2 EBV’s secret weapon to promote cell proliferation

Although EBV-induced cell proliferation itself may not be considered a direct immune evasion mechanism, it becomes relevant due to its interplay with the virus’s escape strategies. EBV’s evasion tactics are elaborately linked to promoting cell proliferation, a process that safeguards cells transformed by the virus from elimination (24) One of EBV’s secret weapons to stimulate cell proliferation is the viral protein EBNA1, capable of binding to the repeat family (FR) element in the oriP region of the viral genome. The binding of EBNA-1 to the FR element is a highly specific interaction that is essential for EBV’s ability to replicate. This binding triggers a cascade of events that culminates in the expression of LMP1, a protein that promotes cell growth. Any disruption of this binding can lead to the inhibition of EBV replication (52, 53).

The oriP region, recognized as the origin of EBV replication, is composed of two distinct functional components: the Dyad Symmetry Element (DS) and the Family of Repeats (FRs). The virus’s unique structural configuration, characterized by the specific interaction with the FR element, stands as a cornerstone of its replicative dynamics (54). This intricate choreography underscores the importance of this interaction as a potent EBV strategy to modulate host cell proliferation (55). By orchestrating alterations in cell proliferation rates, EBV indirectly assists its survival by fostering an environment conducive to viral replication and persistence.

Building upon the foundation of this complex web of interactions, an *in vitro* study aimed to untangle the involvement of EBER1 and EBER2 by investigating the role and mechanism of EBER1-induced cell proliferation (56). Using cellular models, the researchers noted that EBER1 instigates cellular proliferation through the augmentation of mitochondrial activity and the influx of calcium ions (**Figure 1**). Additionally, an investigation utilizing recombinant EBVs containing EBER1 and EBER2 aimed to delineate their individual roles in transforming B cell growth. The findings revealed a significant discrepancy: EBER2 demonstrated a more substantial impact compared to EBER1. This disparity indicates that despite their structural similarities, these proteins distinctly execute diverse functions within latently infected lymphoblastoid cells (57).

The oncogenic potential associated with EBV infection is mainly related to LMP1. In a closely related context, the signaling mechanisms of LMP1, with particular emphasis on its C-terminal domains, instigates a sequence of signaling events facilitated by either tumor necrosis factor receptor (TNFR) associated factors (TRAFs) or TNFR associated death domain proteins (TRADD). This culminates in the activation of critical signaling pathways, including NF-kappa B, JNK, and p38. Consequently, these pathways collectively stimulate growth and survival mechanisms, resulting in an amplification of cellular survival and growth (**Figure 1**) (58).

Throughout the latent phase of EBV infection, the expression of various viral genes defines distinct stages known as latencies, categorized from 0 to III based on observed patterns of gene expression (59). In this cycle, a limited number of proteins are produced, such as EBNAs and LMPs, which perform specific functions associated with EBV infection and its interaction with host cells (59, 60). These biological effects are unique and come to fruition through direct interactions with cellular proteins. For instance, in Burkitt lymphoma cells, EBERs exhibit resistance to IFN-α-induced apoptosis by inhibiting PKR phosphorylation through their direct binding (35).

EBNA1 is a protein consistently expressed in all phases of EBV latency, except in latency phase 0 (16). Its primary role is closely associated with both the preservation of the viral genome and the control of genetic transcription (61). Furthermore, it plays a vital part in maintaining the viral genome, involving itself in DNA replication and the separation of viral chromosomes during cell division (62). Alongside these functions, EBNA1 employs various immune evasion strategies, primarily focused on suppressing MHC class I presentation in the host. This pivotal strategy substantially boosts the virus ability to evade detection by the immune system (63, 64).

EBNA2 assumes the role of a transactivating factor of viral and cellular gene expression. Predominantly expressed during the initial phases of latency, it plays a crucial role in the transition between latency I and latency III (16). Activation of viral and cellular genes triggers the transcription of a cascade of primary and secondary genes, essential for the conversion of normal B cells into immortalized cells (65). This subsequent development drives the proliferation of transformed cells, resulting in uncontrolled growth. Furthermore, EBNA2 demonstrated the ability to interact with other transcription factors involved in the Notch signaling pathway. This pathway, in particular, may be associated with the development of T-cell lymphoma in humans (66).

To better characterize and elucidate the role of EBV in cell growth, it is crucial to understand the specific molecular mechanisms by which EBV proteins, such as LMP1, promote cell growth in both *in vitro* and *in vivo* models. This could allow us to assess latent EBV infection in undifferentiated and differentiating epithelial cells, and answer many questions about this topic that remain unanswered due to the limitations of existing models. *In vitro* cellular models can be used to identify the downstream signaling pathways that are activated by EBV proteins, and animal models of EBV infection can be developed to assess the effects of EBV on cell growth and tumor development.

### 2.3 The art of deception: EBV’s intricate web of immune recognition inhibition

Virus-specific CD8^+^ cytotoxic T lymphocytes (CTLs) play a significant role in the context of viral infections. These cells exhibit a distinct characteristic in which their gene expression profile, specifically related to the encoding of lytic proteins like granzymes, displays significant variations (67). This phenomenon becomes more pronounced in cases of persistent viral infections. For instance, in HTLV-1 infection, the expression levels of genes encoding proteins that mediate cellular cytotoxicity (granzymes, perforin, granulysin) vary depending on the proviral load – the amount of viral genetic material integrated into the host cell’s genome (68). This proviral load acts like a ticking time bomb, replicating alongside the cellular genome and often serving as a potent risk marker for disease development in chronic retroviral infections (69, 70). The persistent nature of EBV infection in B cells suggests a parallel role for EBV-specific CD8+ cytotoxic T lymphocytes (CTLs) in shaping disease outcome. These CTLs may influence the development of EBV-associated malignancies like Burkitt’s lymphoma and Hodgkin’s disease through their immune response to the virus.

The EBV evasion strategy relies on several intricate molecular mechanisms, many of which involve manipulating interactions between the virus and its host at a molecular level. These mechanisms collaboratively establish an environment conducive to EBV survival and replication (64). The EBNA1 protein is characterized by having N and C terminal domains, separated by a unique repeat domain containing glycines and alanines, known as the GAr domain (71). The GAr domain of EBNA1 is intricately linked to its distinctive capacity to evade MHC class I presentation. One of the virus evasion strategies involves proteasomal inhibition. This happens due to the repeat domain within GAr, which nullifies and hampers EBNA1 degradation by the proteasome. Consequently, this inhibition contributes significantly to the virus successful evasion of the immune response (72, 73).

The oncoprotein LMP1, a homologue of TNFR, acts synergistically to inhibit cell death in actively proliferating cells during latency by triggering the NF-Kβ pathway (74). EBV induces the expression of MCL-1 and BFL-1, granting resistance against cellular apoptosis. This mechanism extends the lifespan of the infected host cell, facilitating sustained viral replication (75) Furthermore, as previously stated, EBV miRNAs play a role in regulating cell proliferation, impacting apoptotic processes, and participating in diverse molecular pathways associated with oncogenesis. They stand as pivotal targets for the virus, enabling it to evade the host’s innate and adaptive immune responses (76, 77).

**Table 1** decodes EBV’s escape artistry, revealing the specific molecules it uses to disarm the immune system. These viral strategies go beyond mere illusions - they actively manipulate the cellular environment, influence gene expression, and modulate key signaling pathways to orchestrate a multifaceted evasion strategy. By wielding these molecular tools, EBV effectively slips past the watchful eyes of CTLs, solidifying its place within host cells.

**Table 1:** Mechanisms used by EBV to evade the host’s immune system.

Molecule	Escape mechanism	References
EBNA1	Prevents presentation of the major histocompatibility complex (MHC) class I-restricted EBNA1 epitope to cytotoxic T cells.	(78, 79)
EBNA2	Creates an anti-inflammatory setting by triggering IL-37 and inducing IL-18 receptor expression in B cells. Moreover, it stimulates the induction of PD-L1 to help the virus evade the host’s immune response upon infecting primary B cells.	(80, 81)
LMP1	Downregulates RIG-I signaling pathway by promoting RIG-I degradation dependent on proteasome, allowing the evasion of RIG-I mediated immune responses.	(82)
LMP2	Encodes two transmembrane proteins: LMP2A and LMP2B. LMP2A can reduce MHC class II expression in lymphoblastoid cell lines through Class II transactivator (CIITA) downregulation.	(83)
vIL-10	Impairs NK cell activity in destroying EBV-infected B cells and possessing various immunomodulatory activities by inhibiting cytokine expression. Also, it triggers less STAT3 phosphorylation, reduces anti-inflammatory gene expression, and inhibits M2 polarization in monocytes, contributing to autoimmune reactions in diseases like systemic lupus erythematosus (SLE)	(84, 85)
BNLF2a	Acts as an inhibitor of the Transporter associated with Antigen Processing (TAP), leading to reduced antigen presentation interfering with the recognition of CD8^+^ T cells and thus the detection of EBV-infected cells.	(84)
BPLF1	Contributes to innate immune evasion through interference with Toll-Like receptor signaling, employing deubiquitination of TLR components. Furthermore, it is capable of leading to a decrease in type I IFN production by suppressing cGAS-STING and RIG-I-MAVS pathways.	(86, 87)
BGLF5	Suppresses the expression of several immune components, including TLR9, TLR2, CD1d and HLA molecules, contributing to EBV immune evasion.	(88–90)
gp350	Impairs the immune response by binding to CR2, preventing the CR2-C3d interaction that is important in the link between innate and adaptive immune responses.	(91, 92)
microRNAs	Helps viral escape from the immune response. miR-BART2, for example, targets the stress-induced immune ligand MICB, reducing its expression and thus resulting in escape recognition by natural killer cells.	(93–95)

EBV EBNA1 is one of the molecules that has developed strategies to remain invisible to the immune system by preventing antigen presentation on MHC class I molecules (78). As previously mentioned, EBNA2 functions as a transcriptional activator that manipulates B cell receptor signaling. This manipulation regulates the growth and survival of infected B cells (96). EBNA2 uniquely triggers IL-18 receptor expression in B cells, independently of its interaction with recombination signal binding protein for immunoglobulin kappa J region (RBPJ) (80). IL-18Rα and IL-1R8 forms a complex with IL-37, suppressing pro-inflammatory cytokines and thereby creating an anti-inflammatory environment (97). An anti-inflammatory environment is advantageous to EBV, as the impairment of the immune system facilitates its evasion of the host’s innate and acquired responses (98). Upon infecting primary B cells, EBNA2 is responsible for triggering a substantial increase in the expression of programmed cell death ligand-1 (PD-L1) (81). This heightened PD-L1 induction is a key mechanism employed by EBNA2 to evade the host’s immune response.

LMP1 plays multiple roles in EBV pathogenesis. Notably, it downregulates pro-apoptotic genes, granting it anti-apoptotic potential and contributing to cell survival (99). Additionally, LMP1 disrupts host defenses by evading RIG-I-mediated immune responses, potentially promoting cancer progression (82). RIG‐I is a crucial pattern recognition receptor in the innate immune response against viral infections. It induces cell death in virus-infected cells, triggers production of pro-inflammatory cytokines, and orchestrates other essential antiviral mechanisms (100, 101). LMP1 has been shown to upregulate the expression of PD-L1 as well. This was demonstrated in a study where EBV-positive nasopharyngeal carcinoma (NPC) cell lines displayed higher PD-L1 expression compared to EBV-negative counterparts. Notably, knocking down LMP1 in these EBV-positive cells suppressed PD-L1 expression, further solidifying the regulatory role of LMP1 in this process (102).

*LMP2* is a gene expressed in various EBV-associated diseases, involved in the activation, proliferation and survival of tumor cells, and generates two transcripts: LMP2A and LMP2B (103). LMP2A is capable of reducing MHC class II expression through interference with the E47/PU.1-CIITA pathway (83). Class II transactivator (CIITA) is a master regulator of class II MHC genes and can upregulate class I MHC genes expression (104). By impairing the expression of MHC class II, EBV can avoid its recognition by CD4 T cells, allowing immune escape. EBV also encodes a lytic phase protein called viral interleukin-10 (vIL-10), which shares homology with human IL-10 and can suppress the production of pro-inflammatory cytokines (85).

Expressed during the lytic phase of EBV, the BNLF2a molecule plays a crucial role in immune evasion by interacting with the Transporter Associated with Antigen Processing (TAP) (105). BNLF2a, by inhibiting TAP, exerts direct control over the availability of antigenic peptides for binding to Class I MHC. This mechanism leads to a limitation in antigen presentation on the membrane of the EBV-infected host cell (106). As a consequence, the effective detection capability by CD8^+^ T cells is compromised, granting infected cells the ability to evade a direct immune response. In an *in vivo* study, it was noted that BNLF2a and vIL-10 synergistically collaborate to impact both innate and adaptive immune responses (84).

Another lytic phase protein, BPLF1, employs a multifaceted strategy to evade the immune system. It dampens Toll-like receptor (TLR) signaling by deubiquitinating their components, potentially suppressing innate immune responses (86). Moreover, BPLF1 reduces type I interferon (IFN-I) production by inhibiting the cGAS-STING and RIG-I-MAVS pathways, crucial for detecting viral DNA and activating antiviral defenses (87).

Burkitt lymphoma-associated protein BGLF5 suppresses essential immune components like TLRs and HLA molecules, potentially through enhanced mRNA degradation. This widespread shutdown of cellular gene expression, encompassing HLA class I and II molecules, may significantly impair HLA class I-restricted CD8^+^ T cell recognition (88, 90). Additionally, it inhibits TAP transport, crucial for antigen presentation to CD4^+^ and CD8^+^ T cells (88).These combined strategies enable BGLF5 to effectively silence the immune system and create a favorable environment for EBV replication and survival. Additionally, the gp350 glycoprotein plays a significant role in immune evasion by disrupting viral antigen recognition and B cell activation (107). The extensive evidence presented highlights the sophisticated strategies employed by EBV to evade the host’s immune response. Its arsenal boasts an array of molecules, each contributing to viral persistence and, in some cases, promoting the development of malignancies. Delineating these mechanisms is crucial for unlocking new therapeutic avenues to benefit patients afflicted with EBV-associated diseases.

## 3 Invisible enemy: hiding from the innate immune system

### 3.1 EBV’s cunning tricks to evade the pattern recognition receptors (PRRs)

The process of EBV infection involves several complex interactions with the host’s innate immune system. The initial step includes recognizing pathogen-associated molecular patterns (PAMPs) via a network of pattern recognition receptors (PRRs) (108). Toll-like receptors (TLRs), NOD-like receptors (NLRs), RIG-I-like receptors (RLRs), and C-type lectin receptors (CLRs) are among the main pattern recognition receptors (PRRs) that play a critical role in the initial detection of EBV particles and the initiation of the innate immune response (109).

**Figure 2:**
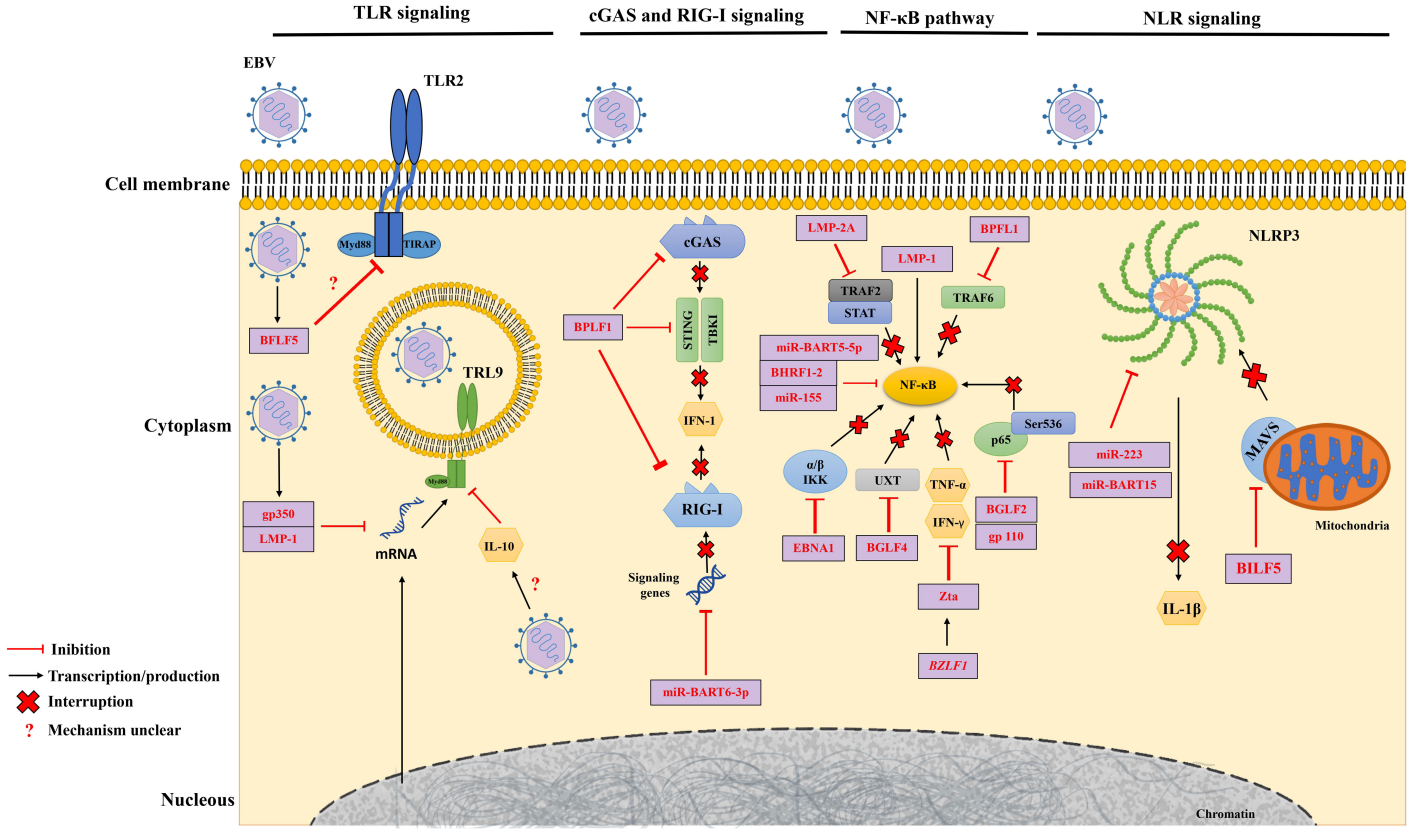
EBV-Mediated Mechanisms of Immune Evasion. EBV evades the immune response by interfering with key molecular pattern receptors (PRRs) and the nuclear factor kappa B (NF-κB) pathway. EBV molecules, proteins and miRNAs play roles in suppressing the activity of Toll-like receptors (TLRs) (left), cyclic GMP-AMP synthase (cGAS) and retinoic acid-inducible gene I (RIG-I) (center left), in the NF-κB pathway (center right) and the NOD-like receptor (NLR) (right). Furthermore, EBV disrupts the production of products resulting from these pathways, directly or indirectly.

In the scenario of the primary EBV infection, TLRs assume a crucial function by facilitating the initial identification of the virus within innate immune cells (110). For example, TLR9 recognizes EBV nucleic acids and activates signaling pathways that lead to the production of pro-inflammatory cytokines, such as type I interferon (IFN-I) or transforming growth factor (TGF), which triggers the antiviral immune response (111). Conversely, EBV employs cunning tactics to slip through the notice of these PRRs, masterfully reshaping the immune response to its advantage (**Figure 2**).

During primary EBV infection, the lytic phase protein, gp350, and the latent phase protein, LMP1, jointly downregulate the TLR9 expression, a pattern recognition receptor that plays an important role in the immune response to the virus. This combined endeavor employs RNA degradation, mRNA reduction, and triggers a TLR9 transcript imbalance through NF-κB activation (112, 113). BGLF5 also acts to reduce TLR2 expression in infected cells, but the exact mechanism is still unknown (114). A study found that individuals infected with EBV had significantly lower levels of TLR9. This decrease in TLR9 levels may be linked to the serum levels of IL-10, an anti-inflammatory cytokine (115).

Transitioning into the latency period, EBV’s strategy shifts gears. During this phase, the virus encodes miRNAs that wield the remarkable ability to intricately regulate gene expression, directly influencing both the course of infection and the manipulation of the host’s immune system (116). A key role of these miRNAs emerges as their capacity to effectively suppress TLR expression within infected cells (117). This strategic suppression of TLRs allows EBV to regulate the inflammatory response and evade detection by innate immune cells. The complex interplay of these events reveals EBV’s masterful ability to navigate the immune landscape.

EBV is also able to suppress innate immune signaling mediated by two other receptors, Cyclic GMP-AMP Synthase (cGAS) and RIG-I (87). During lytic replication, EBV protein BPLF1, which has deubiquitinase (DUB) activity, inhibits the production of IFN-1 stimulated by cGAS and RIG-I by acting on the stimulator of interferon genes (*STING*) and TANK-binding kinase 1 (TBK1) signaling pathways (87). The RIG-1 pathway can also be inhibited during the latency phase through overexpression of the EBV miRNA, miR-BART6-3p, which prevents the expression of signaling genes for this receptor (118). These complex strategies showcase EBV’s adeptness in manipulating host defenses for its advantage.

EBV also exploits receptors from the nucleotide-binding oligomerization domain (NOD)-like receptors (NRL) family as part of its evasion tactics against the immune response. Because these receptors have nucleotide-binding and oligomerization domains, they facilitate the formation of inflammasomes (119). A notable example of an NLR is the pyridine-containing domain 3 (NLRP3), in which its activation induces the production of cytokines such as interleukin-1β (IL-1β) (119). Increased levels of EBV miR-223 and miR-BART15 lead to the suppression of NLRP3 production and, as a result, hinder the generation of IL-1β (120). In addition to miRNAs, the lytic protein BILF1 acts to block NLRP3 activation by selectively removing mitochondrial antiviral signaling protein (MAVS) from mitochondrial membranes (121). By limiting the inflammatory capacity of NLRP3, EBV ends up suppressing the host’s innate immune activity (**Figure 2**).

The interaction between EBV and PRRs is the initial and critical phase in determining whether the virus is controlled by the host or escapes the immune system. TLR9 can restrict EBV lytic replication, but the latent viral protein LMP1 can suppress TLR9 transcription. EBV can also inhibit the activity of other receptors, such as cGAS and RIG-I, impairing the innate immune response. It remains unknown how EBV evades the immune system through additional receptors, such as CLRs. This complex molecular dance highlights EBV’s ability to manipulate the immune system to its own benefit.

### 3.2 EBV’s elusive ballet beyond NF-κB: unraveling immunity’s “locomotion”

EBV has artfully devised an escape plan from the host’s immune clutches. Among its ingenious tactics is the deft manipulation of the NF-κB transcription factor pathway. NF-κB, a pivotal commander in the realm of immune and inflammatory responses, assumes a vital role in triggering genes that coordinate the production of pro-inflammatory cytokines and cell adhesion molecules (122). Through this cunning orchestration, EBV unfurls a veil of invisibility, slipping past detection and quelling the host’s immune retort. This scenario serves as a mere glimpse into the multifaceted ways this virus can artfully outwit our immune defenses (123).

During EBV infection, activation of the canonical NF-κB pathway begins through stimulation by the latent virus protein LMP1 (124, 125). EBV exhibits a dualistic interplay with the NFκB signaling pathway. During its latent state, EBV induces NF-κB activation to promote cell proliferation and survival. However, during its lytic replication cycle, EBV blocks NF-κB signaling, which inhibits cell proliferation and promotes apoptosis. This dual regulation of NF-κB by EBV allows the virus to evade the host immune system and establish a persistent infection (108).

During the course of lytic infection, EBV encodes a variety of proteins that downregulate NF-κB signaling, promoting viral DNA replication. BPLF1 deubiquitinates TNF receptor associated factor 6 (TRAF6) protein to suppress the NF-κB pathway (126). This prevents TRAF6 from being degraded and allows it to activate NF-κB. The tegumentary protein BGLF2 prevents NF-κB activity by blocking p65 phosphorylation at Ser536 and its nuclear translocation (127). EBV glycoprotein 110 also interacts with the p65 subunit of NF-κB, suppressing its phosphorylation and nuclear translocation (128).

Similarly, the Zta protein encoded by the viral gene *BZLF1* inhibits the transcription of TNF-α and IFN-γ by binding to the TNF-α promoter, attenuating the NF-κB response (13, 129). Another EBV lytic protein, BGLF4, restricts NF-κB activation by phosphorylating the ubiquitously expressed transcription coactivator protein (UXT), which reduces the interaction between UXT and NF-κB (130). BGLF4 is also involved in escaping the innate immune response, suppressing IRF3 (Interferon Regulatory Factor 3) transactivation activities (131). This is achieved through phosphorylation of multiple sites on IRF3, which leads to suppression of IFN production and therefore may facilitate EBV replication (131).

Inhibition of NF-κB activation also occurs during the latency period. The EBV latency protein, LMP2A, inhibits NF-κB in carcinoma cells by down-regulating transcriptional pathways such as TRAF2 and the signal transducer and activator of transcription (STAT) (132, 133). EBNA1 functions as a suppressor of the canonical NF-κB signaling pathway by impeding the phosphorylation of both the α/β kinase complex (α/β IKK) and p65. Additionally, EBNA1 augments the functionality of the Protein Activator 1 (AP-1) transcription factor (134, 135).

EBV miRNAs, BHRF1-2 and miR-155, also can inhibit NF-κB activation, suppressing innate immunity against latent EBV infection (136, 137). In NPC, NF-κB activation induces EBV miR-BART5-5p expression, which subsequently suppresses LMP1 expression, resulting in autoregulatory modulation of NF-κB and sustaining EBV latency (114). EBV BHRF1 miRNAs exhibit a unique expression pattern, detectable in both cells harboring stage III latent EBV infection and those undergoing lytic replication within EBV-positive tumors (138, 139). These miRNAs play a pivotal role in EBV-associated oncogenesis by wielding a double-edged sword: promoting cell proliferation and simultaneously inhibiting apoptosis. This functional duality is evident in studies demonstrating increased apoptosis upon BHRF1 miRNA knockdown in EBV-infected B cells (140, 141).

EBV BHRF1 miRNAs exhibit a unique expression pattern, detectable in both cells harboring stage III latent EBV infection and those undergoing lytic replication within EBV-positive tumors (138, 139). These miRNAs play a pivotal role in EBV-associated oncogenesis by wielding a double-edged sword: promoting cell proliferation and simultaneously inhibiting apoptosis. This functional duality is evident in studies demonstrating increased apoptosis upon BHRF1 miRNA knockdown in EBV-infected B cells.

It is relevant to mention that *BHRF1* and *LMP1* are strongly linked to malignant transformations related to EBV and represent prime targets for the development of a vaccine (142). However, genetic variability and mutations in both genes require deeper understanding and strategies to overcome these issues in vaccine development.

### 3.3 Natural killer cell dynamics and receptor repertoire in the battle against EBV

Natural killer cells (CD3^-^CD56^+^) represent one of the first and main lines of defense of innate immunity against EBV (143). NK cells can be divided into two main subpopulations based on the expression of the CD56 (adhesion glycoprotein) and CD16 (IgG receptor) molecules, namely CD56^dim^ CD16^bright^ NK cells and CD56^bright^ CD16^dim/neg^ NK cells (143). NK cells still have a variety of receptors on their surface that can both activate and inhibit their cytotoxic activity. NK cell activating receptors are a group of cell-surface receptors that recognize and bind to specific ligands on the surface of target cells. These receptors trigger NK cell activation and release of cytotoxic molecules that kill the target cells (144). The group of receptors responsible for activating NK cells encompasses several key components, notably the killer immunoglobulin-like receptors (KtIRs), DNAX coactivation/adhesion activating molecule (DNAM-1), NKG2D receptors, and the subset of natural cytotoxic activation receptors (NCRs), which comprises NKp30, NKp44, and NKp46 (145).

Upon infection with EBV, NK cells can either secrete pro-inflammatory cytokines, such as IFN-γ and TNF-α, or directly eliminate virus-infected cells (146). In the acute phase of infectious mononucleosis, a distinctive subset of early-differentiated NK cells known as CD56^dim^ NKG2A^+^ immunoglobulin-like receptor^-^ (KIR^-^) cells proliferate and accumulate, with their number increasing up to fivefold (147). These CD56^dim^ NKG2A^+^ KIR^-^NK cells mainly target EBV-infected B lymphocytes with cytotoxic properties. Additionally, it is pertinent to highlight that the inhibitory responses associated with NKG2A^+^ cells and the absence of activation related to NKG2C^+^ cells are correlated with the pathogenesis of Hodgkin lymphomas and non-Hodgkin lymphomas associated with EBV (148).

The proliferation and accumulation of NK cells in IM is thought to be a defense mechanism against the virus (149). NK cells are able to recognize and kill EBV-infected cells through a variety of mechanisms, including the release of cytotoxic molecules and the induction of apoptosis (150). The activation of NK cells is also thought to be important for the production of pro-inflammatory cytokines, mainly IFN-γ, which can help to control the spread of the virus (151). The role of NK cells in IM is an area of active research. Further studies are needed to understand how these cells are activated and how they contribute to the immune response against EBV.

However, in an attempt to evade the host’s immune response, EBV can reduce or inhibit the expression of the class I major histocompatibility complex (MHC I), thus preventing recognition and elimination by CD8^+^ T lymphocytes (152). However, this mechanism inadvertently triggers the activation of the cytotoxic response of NK cells, since inhibitory receptors on NK cells specifically recognize class I MHC molecules (153). In addition, the cytotoxic function of NK cells can be activated through the recognition, by KIRs and NCRs receptors, of factors associated with the stress generated by EBV infection (152).

Therefore, the activation of NK cells by EBV infection is a complex process that involves both the inhibition of MHC I expression and the recognition of stress-induced ligands. Additional studies are needed to elucidate the precise mechanisms involved in this process, as this knowledge could be used to develop new strategies for the treatment of EBV-associated diseases.

In spite of the complex and effective immunosurveillance undertaken by NK cells, EBV employs a range of alternative strategies to attenuate and circumvent this immune counteraction. In the context of its lytic replication, EBV utilizes the *BCRF1* and *BNLF2a* genes to encode vIL-10, an anti-inflammatory cytokine that decreases the production of IFN-γ and IL-2 and interferes with NK cell cytotoxicity. By inhibiting the expression of these cytokines, vIL-10 can help EBV-infected cells evade NK cell killing (84). This mechanism is thought to be important for EBV to establish and maintain a latent infection.

Cells infected with EBV, in the late lytic phase, escape the immune system mainly by two mechanisms. First, the viral product BHRF-1, a homologue of vBcl-2, inhibits the activity of the pro-apoptotic protein BAX, preventing the activation of the intrinsic apoptotic pathway (154, 155). Second, the lytic cycle regulator gene *BZLF1* can induce the expression of NKG2D ligands on the cell surface, which can be recognized by NK cells and lead to their activation and killing of the infected cells. However, BHRF1 can inhibit the expression of NKG2D ligands, thereby protecting EBV-infected cells from NK cell killing (156, 157). The BHRF1 protein may also play a protective role against CD8^+^ and CD4^+^ T cells, but this possibility needs further studies (156).

In EBV-related epithelial malignancies, such as NPC and EBV-associated gastric carcinoma (EBVaGC), the LMP2A has been shown to upregulate the expression of the *F3* gene, through the activation of the P13/AKT signaling pathway (158). This pathway promotes platelet aggregation and inhibits the antitumor function of NK cells (158). LMP2A can also reduce the expression of molecules on B cells that interact with NKG2D receptors, which are responsible for activating the cytotoxic response of NK cells. This neutralization of B cell recognition by NK cells allows EBV-infected B cells to evade the immune system (159). These findings suggest that LMP2A is a key mediator of EBV-induced tumorigenesis. Targeting LMP2A may be a promising strategy for the development of novel therapies for EBV-related malignancies.

Within the latent phase occurring in B cells, the EBV latent viral gene *EBNA1* suppresses the cytotoxic response of NK cells by down-regulating the expression of NKG2D and c-Myc, a key protein of apoptosis (160). Furthermore, EBV display its capability to suppress the cytotoxic responsiveness of NK cells through the encoding of two miRNAs, pri-miR-BART2 and miR-BART2-5p. These miRNAs assume a critical function in the inhibition of mRNA translation associated with the B sequence found within the main complex of MHC I, specifically the MICB. Notably, MICB represents an indispensable ligand for the NKG2 receptor (93).

The relationship between EBV and NK cells is a multifaceted and constantly evolving area of research. It unveils the virus complex tactics for eluding the immune system. EBV employs various strategies, such as generating anti-inflammatory cytokines, impeding apoptosis, and diminishing the cytotoxicity of NK cells through miRNAs. These discoveries point toward a potential avenue for therapeutic development—targeting specific EBV components like the LMP2A protein or miRNAs. This approach holds promise in the pursuit of treatments for EBV-related malignancies.

### 3.4 The art of evasion: how EBV escapes from the nature’s vigilant virus hunters - myeloid cells

One of the key players in innate immunity is the myeloid cell lineage. Myeloid cells are found throughout the body, and they play a variety of roles in the immune response, including phagocytosis, antigen presentation, and the production of many immune mediators (161, 162). In the context of EBV infection, myeloid cells play a critical role in mounting an antiviral response.

Myeloid cells, such as phagocytic and antigen-presenting cells, actively participate in the antiviral response against EBV (110). For example, macrophages act as sentinels, phagocytosing EBV and activating specific responses, while dendritic cells capture viral antigens and coordinate T cell activation (163, 164). In doing so, they construct a fundamental first line of defense against EBV infection, thereby facilitating a more potent adaptive response primed to curtail the virus. However, EBV also seeks mechanisms to evade this control.

EBV can indirectly suppress the response of NK, T and phagocytic cells by stimulating myeloid-derived suppressor cells (MDSCs) through the LMP-1 protein (165, 166). The LMP-1 can induce the release of cytokines and growth factors, such as IL-6, GM-CSF, and IL-1β. These immune mediators drive the expansion of MDSCs, which subsequently inhibit other myeloid cells through the generation of reactive oxygen species (ROS), L-arginine depletion, and downregulation of the NK receptor NKp3 (166, 167).

Beyond its interaction with epithelial cells, EBV’s spectrum of influence extends to monocytes, which it can lytically infect, leading to significant repercussions (168). Consequently, EBV-infected monocytes show an average reduction of 50% in their phagocytic function and inhibition of NF-κB activation in these cells (168, 169). Furthermore, the virus subverts monocyte survival by diminishing autophagy, intracellular ROS levels, and mitochondrial biogenesis within these cells (170). The viral cytokine vIL-10 further suppresses the anti-inflammatory phenotype of monocytes by reducing phosphorylation of STAT3 and the scavenger receptor CD163 (85). Adding another layer to its repertoire of immune evasion tactics, EBV’s lytic protein Zta steps into the spotlight by activating Suppressor of Cytokine Signaling 3 (*SOCS3*). This activation curbs the production of IFNα in monocytes, constituting yet another facet of EBV’s elaborate immune escape strategy (171).

Although there is no evidence that EBV infects DCs during primary infection, *in vitro* model studies have demonstrated that EBV can evade the immune response of DCs through different ways. For example, one study found that EBV can inhibit the phenotypic differentiation of DCs derived from umbilical cord blood monocytes and induce their apoptosis in a caspase-dependent manner, with activation of the mitochondrial pathway (172). This suggests that EBV can prevent DCs from maturing and becoming fully functional, thereby limiting their ability to activate the adaptive immune response.

Another study found that EBV reduces the production of type I interferon (IFN) by plasmacytoid DCs through the latency proteins EBNA3A and EBNA3C (173). IFN is a key cytokine that plays a role in antiviral immunity. By reducing IFN production, EBV can make it more difficult for the immune system to fight the virus. Finally, during EBV-associated MALT lymphoma, there is a significant IL-10-mediated loss of plasmacytoid DCs, resulting in immune dysregulation (174). IL-10 is a cytokine that has anti-inflammatory effects. The loss of plasmacytoid DCs in this setting could contribute to the development of MALT lymphoma.

EBV is capable of infecting neutrophils and inducing apoptosis in these cells, possibly through the Fas/Fas ligand (FasL) system (175, 176). The mechanism by which EBV triggers apoptosis in neutrophils is not fully understood. However, one possibility is that EBV-infected cells may express FasL or a similar molecule on its surface. When FasL on the surface of an infected neutrophil binds to Fas on another neutrophil, it triggers apoptosis in both cells. By killing neutrophils, EBV can reduce the number of cells that are able to fight the virus.

The available scientific literature highlights the critical role of myeloid cells in the innate immune response against EBV infection. These cells are well-documented for their diverse functions, including phagocytosis and antigen presentation, which serve to initiate a robust defense against the virus. However, EBV’s impact extends beyond this, as it interferes with dendritic cells, hindering their differentiation and promoting apoptosis through mechanisms that require deeper exploration. Additionally, EBV reduces the production of type I interferons by plasmacytoid DCs, impairing antiviral immunity. In cases of EBV-associated MALT lymphoma, the virus triggers the loss of plasmacytoid DCs, contributing to immune dysregulation. Further studies are essential to comprehensively understand the involvement of myeloid cells in EBV immunity and develop effective strategies to counter the virus evasion tactics.

## 4 Dancing in shadows: EBV’s artful escape from acquired immune response

**Figure 3:**
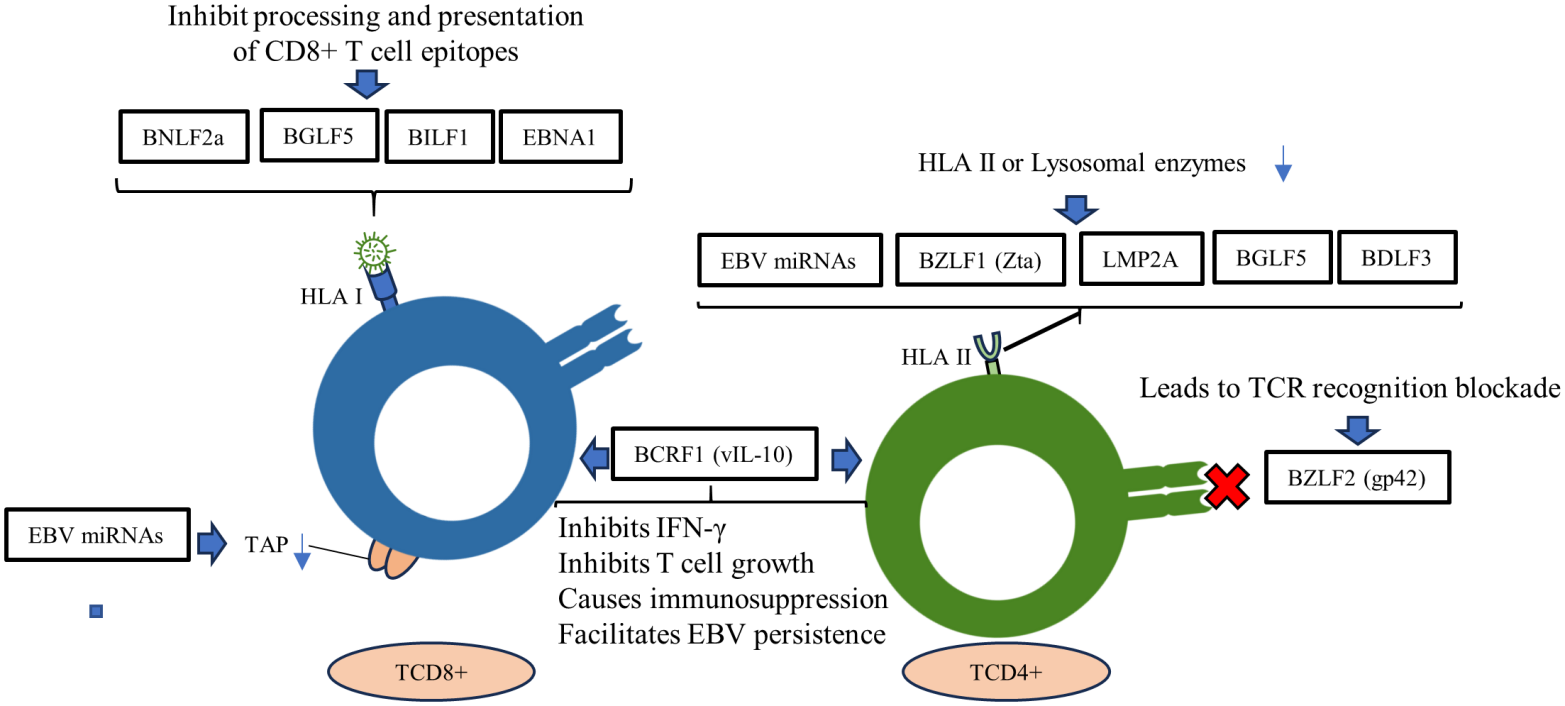
Roles of EBV genes and proteins in evading the adaptive immune response. EBV miRNAs negatively regulate the transporter complex associated with antigen processing (TAP), affecting antigen presentation by MHC class I. EBV lytic and latency proteins disrupt HLA I and II presentation in host cells, inhibiting the activation of CD4^+^ and CD8^+^ T cells.

The interplay between innate and acquired immunity is crucial for an effective immune response. In this context, EBV employs strategies to thwart the mechanisms of acquired immunity, enabling it to evade detection and destruction. Although our understanding of how EBV evades the acquired immune system is incomplete, one important strategy involves disrupting the presentation of HLA I and II antigens. This leads to a decrease in the expression of these molecules on the cell surface, which in turn impairs the activation of CD4^+^ and CD8^+^ T cells. This interference prevents the coordinated response that is necessary for fighting EBV infection (177, 178) (**Figure 3**). This is probably the most important mechanism that EBV uses to evade the acquired response, considering the indispensableness of the HLA system in regulating the immune response (179).

EBV’s remarkable ability to evade the immune system relies heavily on its disruption of crucial interactions between different components. One such key disruption involves the gp350 glycoprotein, which wreaks havoc on the intricate interplay between B cells and the complement system. The gp350 glycoprotein contributes significantly to immune evasion by obstructing the interaction between B cells and the complement system. It does so by binding to complement receptor 2 (CR2) on the surface of B cells. In regular circumstances, CR2 engages with the C3d receptor, a vital part of the complement system (101). However, gp350 interrupts this process by directly binding to CR2, preventing the subsequent binding of C3d. As a consequence, B cells lose their ability to properly recognize and respond to viral antigens, impairing the activation of the adaptive immune system (92).

Moreover, EBV miRNAs have been shown to downregulate the transporter associated with antigen processing (TAP) complex, which affects MHC class I antigen presentation (180). This is done by targeting the *TAP1* gene, which is essential for the transport of peptides into the endoplasmic reticulum. As a result, fewer peptides are available to be loaded onto MHC class I molecules, which are then displayed on the cell surface (181). This makes it more difficult for T cells to recognize and kill infected cells.

In addition to *TAP1*, EBV miRNAs can also target the *HLA II* gene expression and lysosomal enzymes that are involved in proteolysis and epitope presentation. This further reduces the presentation of MHC class II antigens on the cell surface, impairing T cell activation (182) (**Figure 3**). The combined effect of these mechanisms is a reduction of T cell immune surveillance, allowing EBV to evade the immune system and persist in the body.

EBV can also evade the immune response of CD8 T cells by secreting the viral proteins BNLF2a, BGLF5 and BILF1 (183). These proteins are important for EBV escape in all three phases of the lytic cycle: immediate-early (IE), early (E), and late (L). BNLF2a is an IE protein that inhibits the function of the TAP complex. The TAP complex is responsible for transporting peptides into the endoplasmic reticulum, where they are loaded onto MHC class I molecules.

By inhibiting the TAP complex, BNLF2a reduces the number of peptides that are available to be loaded onto MHC class I molecules. BGLF5 is a protein that degrades mRNA encoding MHC class I molecules. This reduces the number of MHC class I molecules that are produced by the cell. BILF1 is a protein that interacts with the MHC class I molecules and prevents them from being displayed on the cell surface (183, 184).

The successful elimination of pathogens depends critically on the complex communication and interdependence between the innate and adaptive immune systems. In this context, TLRs play a pivotal role as sentinels of the immune system. TLRs recognize pathogen-associated molecular patterns and trigger signaling pathways that directly combat the threat or orchestrate the adaptive immune response (185). EBV protein BGLF5 interferes with this vital process by reducing TLR9 levels through RNA degradation, effectively hampering the host’s innate response (89). A study demonstrated a significant re-establishment of key immune-related molecules—such as TLR2, HLA class I/II, and CD1d—when the expression of BGLF5 was silenced in reactivated Akata BL cells (90).

In summary, EBV employs a multi-pronged strategy involving the viral proteins BNLF2a, BGLF5, and BILF1 to effectively evade CD8 T cell immune responses at various stages of its lytic cycle, ultimately impairing the presentation of viral antigens to the immune system. However, further investigation is needed to understand how BGLF5-mediated reduction of TLR9 affects the adaptive immune system overall response. Additionally, exploring the downstream effects of BGLF5 interference on specific adaptive immune pathways could provide crucial insights into its role in impairing the presentation of viral antigens to the immune system during EBV’s lytic cycle.

To evade the immune response of CD4^+^ T cells, EBV uses the Zta transcription factor, which is encoded by the *BZLF1* gene. The Zta transcription factor inhibits the expression of MHC class II molecules by suppressing the activity of the Class II transactivator (CIITA). CIITA is essential for the transcription of MHC class II genes (116, 124).

Alongside the *BZLF1* gene, further investigations have highlighted the engagement of *LMP2A*, *BGLF5*, and *BDLF3* in diminishing the expression of MHC class II. Furthermore, a different facet involves the EBV glycoprotein gp42, which is derived from the *BZLF2* gene. This glycoprotein exerts an inhibitory effect on the antigen-specific activation of T helper cells. This is attributed to its capacity to bind with HLA class II molecules, subsequently resulting in the blockade of TCR recognition (83, 88, 184, 186). Another EBV glycoprotein, gp150, which is expressed during the late phase of the lytic cycle, forms an immune-evasive barrier on infected cells. This glycoprotein, through its glycosylation, inhibits the surface presentation of antigens by HLA classes I and II, as well as by non-classical lipid-presenting CD1d molecules (187).

In addition to the previously mentioned factors, EBV employs other strategies to evade detection by acquired immune response. One key strategy involves vIL-10, a viral homologue of human IL-10 encoded by the *BCRF1* gene. vIL-10 plays a crucial role in suppressing T-cell responses by inhibiting IFN-γ production, hindering T-cell growth, and inducing immunosuppression, thereby supporting viral persistence (188, 189). A study reported that vIL-10 can lead to three main outcomes that are favorable to EBV: modulation of cytokine responses, interference with CD4+ T cell activity and can also prevent NK cell-mediated destruction of infected B cells (84).

Furthermore, another intriguing mechanism employed by EBV centers around EBNA1, which has the ability to inhibit its own presentation to MHC class I, primarily through the Gly-Ala repeat domain (73, 106). This multifaceted immune evasion strategy is crucial for EBV’s ability to persist within the host, as it effectively disrupts various facets of the immune response.

EBV has developed mechanisms to inhibit several other host molecules that are involved in the cellular response. For example, the EBV BPLF1 protein has the ability to regulate cellular signaling pathways, inhibiting type-I IFN responses through TRIM25 autoubiquitination and functional inactivation of the RIG-I signalosome (87). EBV BGLF2 is another protein that suppresses host interferon signaling, recruiting enzymes to remove the phosphate group from STAT1 and redirecting STAT2 for degradation (190).

EBV BHRF1 protein decreases type I IFN induction by impairing mitochondrial dynamics, stimulating mitophagy through interaction with Beclin 1, which is essential in the regulation of autophagy (157). Another EBV protein capable of modulating host IFN-mediated immune responses is BILF4 (LF2), which interacts with IRF7 central inhibitory association domain, resulting in the inhibition of the dimerization of IRF7 and suppression of IFN-α production (191).

EBV employs a complex array of mechanisms to evade the acquired immune response, which is crucial for its persistence within the host. While these mechanisms shed light on the virus intricate strategies, it is important to note that our understanding of EBV’s evasion tactics remains incomplete, emphasizing the need for ongoing research to uncover additional mechanisms and develop more effective strategies for combating this persistent pathogen.

## 5 Conclusion remarks

In the captivating dance of immune evasion, EBV emerges as the cunning choreographer, orchestrating a complex symphony of strategies that tip the scales in its favor. From initial infection to periods of latency, EBV employs a range of tactics: it thwarts apoptosis, spurs cell proliferation, dampens NK and myeloid cell activity, reduces PRRs and HLA expression, fosters an anti-inflammatory milieu by suppressing cytokine expression, and disrupts key host immune recognition molecules.

This review brings together crucial insights into EBV’s crafty immune escape maneuvers, unveiling the intriguing complexities within its molecular arsenal. While extensive research has shed light on many aspects, some questions linger in the shadows. For example, how does EBV induce cancer in diverse tissues? What mechanisms underlie EBER1 ability to stimulate cell growth by manipulating mitochondrial activity and calcium dynamics? Furthermore, how do EBV’s proteins and miRNAs synergize to facilitate cell proliferation and immune evasion? As we navigate this labyrinth of EBV’s immune subversion, the scarcity of answers looms large.

The absence of adequate *in vitro* and *in vivo* models further obscures our quest for clarity, limiting our understanding of EBV’s immune evasion strategies and their consequences. As we endeavor to illuminate these shadowy corners of knowledge, we are reminded of the vital importance of research aimed at uncovering EBV’s immune escape mechanisms, especially regarding acquired immunity. It is in these uncharted territories that potential therapies may emerge—therapies that could disrupt EBV’s elaborate evasion mechanisms and potentially transform the prognosis of patients with EBV-associated cancers. In essence, our journey through this complex choreography is far from complete, and we stand poised to unravel the secrets that the mastermind of immune escape continues to guard.

## Author contributions

JS: Writing – original draft, Writing – review & editing. CA: Writing – original draft, Writing – review & editing. GS: Writing – original draft, Writing – review & editing.
